# Femora from adults with type 1 or type 2 diabetes have lower bone strength and smaller hip geometry

**DOI:** 10.1093/jbmrpl/ziaf127

**Published:** 2025-07-30

**Authors:** Shannon R Emerzian, Fjola Johannesdottir, David C Lee, I-Hsien Wu, Surya Vishva Teja Jangolla, Marc Gregory Yu, Hetal S Shah, George L King, Tony M Keaveny, Klaus Engelke, Elaine W Yu, Mary L Bouxsein

**Affiliations:** Department of Orthopedic Surgery, Center for Advanced Orthopedic Studies, Beth Israel Deaconess Medical Center, Boston, MA 02215, United States; Department of Orthopedic Surgery, Harvard Medical School, Boston, MA 02115, United States; Department of Orthopedic Surgery, Center for Advanced Orthopedic Studies, Beth Israel Deaconess Medical Center, Boston, MA 02215, United States; Department of Orthopedic Surgery, Harvard Medical School, Boston, MA 02115, United States; O.N. Diagnostics, Berkeley, CA, 94704, United States; Research Division, Joslin Diabetes Center, Boston, MA 02215, United States; Research Division, Joslin Diabetes Center, Boston, MA 02215, United States; Department of Internal Medicine, Harvard Medical School, Boston, MA, 02115, United States; Research Division, Joslin Diabetes Center, Boston, MA 02215, United States; Department of Internal Medicine, Harvard Medical School, Boston, MA, 02115, United States; Research Division, Joslin Diabetes Center, Boston, MA 02215, United States; Department of Internal Medicine, Harvard Medical School, Boston, MA, 02115, United States; Research Division, Joslin Diabetes Center, Boston, MA 02215, United States; Department of Internal Medicine, Harvard Medical School, Boston, MA, 02115, United States; O.N. Diagnostics, Berkeley, CA, 94704, United States; Departments of Mechanical and Bioengineering, University of California, Berkeley, Berkeley, CA, 94720, United States; Institute of Medical Physics, University of Erlangen-Nürnberg, Erlangen, 91052, Germany; Endocrine Unit, Department of Medicine, Massachusetts General Hospital and Harvard Medical School, Boston, MA 02114, United States; Department of Orthopedic Surgery, Center for Advanced Orthopedic Studies, Beth Israel Deaconess Medical Center, Boston, MA 02215, United States; Department of Orthopedic Surgery, Harvard Medical School, Boston, MA 02115, United States; Endocrine Unit, Department of Medicine, Massachusetts General Hospital and Harvard Medical School, Boston, MA 02114, United States

**Keywords:** type 1 diabetes, type 2 diabetes, bone structure, BMD, QCT, bone strength, finite element analysis

## Abstract

The increased hip fracture risk in individuals with type 1 (T1D) and type 2 (T2D) diabetes is not explained by areal BMD (aBMD), indicating that diabetes increases fracture risk through mechanisms independent of aBMD. To investigate, we used QCT to compare femoral strength, volumetric BMD (vBMD), and geometry in cadaveric femora from older adults with T1D (*n* = 23; 13 female) and T2D (*n* = 21; 11 female) to controls of similar age, sex, and race (*n* = 19; 11 female). While aBMD and vBMD measures were similar across groups, femoral strength was lower in the diabetic groups compared to controls. Geometric strength, based on external bone shape, was lower in T1D (−15%, *p* = .001) and T2D (−12%, *p* = .014) compared to controls. When combining geometry and density, femoral strength was significantly lower in T1D (−19%, *p* = .044). The strength-to-density ratio was also lower in T1D and T2D (*p* ≤ .013), indicating greater skeletal fragility in the diabetic groups beyond what is predicted by BMD. Diabetic groups had smaller bone size, including lower femoral neck volume (−8%, *p* ≤ .030), neck cross-sectional area (CSA) (−8%, *p* ≤ .030), and trochanter CSA (−7%, *p* ≤ .010). These findings suggest that lower femoral strength and smaller geometry contribute to elevated fracture risk in diabetes, warranting further study in larger populations.

## Introduction

Diabetes mellitus is associated with an increased risk of fracture compared to those without diabetes, with a 5-fold increased risk for hip fracture in type 1 diabetes (T1D) and a 30%-70% increased risk of hip fracture in type 2 diabetes (T2D).^1^^,^^2^ Moreover, mortality following hip fracture is greater in patients with diabetes.^3^ To optimize interventions that reduce fractures in the aging diabetic population, an improved understanding of the factors that contribute to elevated fracture risk is critical.

The elevated fracture risk in individuals with diabetes is influenced by multiple factors, with the primary contributors likely being an increased risk of falls and lower bone strength. However, increased fall risk only partially explains the increased fracture risk in those with diabetes.^4^ Factors that increase skeletal fragility in diabetes are not completely understood. In individuals with T1D, increased fracture risk is only partially explained by lower bone mass, as lower areal BMD (aBMD) measured by DXA explains some, but not all, of the observed risk.^5^ Conversely, individuals with T2D often exhibit normal or elevated aBMD yet remain at increased risk of fracture.^5^ This suggests factors beyond aBMD contribute to skeletal fragility in both T1D and T2D.

Quantitative computed tomography imaging can assess several key contributors to skeletal fragility, including bone strength, density, and morphology (geometry and shape). For example, finite element analysis (FEA) of clinically acquired QCT scans has been used to estimate bone strength.^6^ This technique, also known as biomechanical computed tomography (BCT) analysis,^6^ provides estimates of bone strength that predict femoral failure load better than DXA aBMD or QCT measures of bone density and volume^7^ and predicts hip fracture independently of aBMD.^6^^,^^8^ While the use of BCT-based bone strength has been utilized to evaluate the efficacy of osteoporosis interventions in several trials of osteoporosis treatments,^9^^,^^10^ this technique has yet to be utilized to evaluate diabetic populations compared to non-diabetic controls. Furthermore, QCT data of bone density and morphology at the hip are limited in T1D, with only 1 study in older adults (>50 yr old)^11^ and 2 small studies *(n* = 17) in a single a population of young and middle-aged men (age range 19-48 yr).^12^^,^^13^ These studies using QCT in T1D have shown that in older adults with T1D, those who were diagnosed with T1D before age 15 as well as those with microvascular complications had unfavorable bone outcomes at the femoral neck, such as smaller cortical cross-sectional area (CSA), compared to matched controls.^11^ Studies using QCT at the hip in T2D have shown that older adults (>65 yr) with T2D have greater femoral neck vBMD^14^^,^^15^ compared to age- and sex-matched non-diabetic controls. However, individuals with T2D may have bone geometric deficits, such as impaired femoral neck geometry.^15^^,^^16^ Additionally, in a study of 98 men and women (ages 38-88 yr), those with T2D had a similar ratio of estimated fall force to flexural rigidity, a simplified estimate of bone strength, at the hip and spine compared to non-diabetic controls, suggesting that improvements in bone strength may be offset by higher loads upon falling.^15^ Importantly, no studies to date have investigated proximal femoral bone mass or morphology in a cohort of older adults with T1D and T2D compared to non-diabetic controls.

Thus, our objective was to identify skeletal factors that may contribute to increased hip fracture risk in diabetes by using QCT images. We used QCT and BCT to assess bone strength, mass, and morphology in cadaveric femora from older adults with T1D, T2D, and non-diabetic controls. The primary outcome of this study was to compare BCT bone strength, which we hypothesized would be lower in the diabetic groups (T1D, T2D) compared to non-diabetic controls. Further, we investigated whether there were differences in QCT measures of proximal femoral BMD and morphology between diabetic and control groups.

## Materials and methods

### Study population and specimen collection

Post-mortem, whole femora were donated to the Joslin Medalist Study for the T1D group (*n* = 23), and non-diabetic control femora of similar age, race, and sex were obtained from commercial tissue banks (The National Disease Research Interchange; Anatomy Gifts Registry), as previously published.^17^ For the T2D (*n* = 21) group, femora were also obtained from commercial tissue banks (The National Disease Research Interchange; Anatomy Gifts Registry). Diagnosis of T2D was self-reported by tissue donors.

While age at death, cause of death, height, weight, and BMI were available for all donors, extensive medical history including diabetes disease duration and presence of complications was only available in the T1D cohort from the Joslin Medalist Study, the details of which have been extensively described elsewhere.^18^ In brief, participants in the Medalist Study are Caucasian with 50 or more years of T1D with documented insulin dependence. Study visits were performed at the Joslin Diabetes Center in Boston, MA, between the years of 2005 and 2021, with an average of 7.6 yr antemortem (range: 0-16 yr). The Joslin Committee on Human Studies approved the study protocol, and informed consent was obtained from all subjects prior to participation in the study. During the study visit, individuals with long-duration T1D were evaluated for height, weight, HbA1c, and microvascular disease (ie, neuropathy, retinopathy, and nephropathy). HbA1c was determined by HPLC (Tosoh G7 and 2.2). Peripheral neuropathy was assessed using the Michigan Neuropathy Screening Index, and a score ≥2 was considered positive.^19^ An albumin-to-creatinine ratio >30 mg/g and an estimated glomerular filtration rate <45 mL/min per 1.73 m^2^ was considered positive for nephropathy.^20^ Diabetic retinopathy was diagnosed using 7-standard field fundus photography and graded according to the Early Treatment Diabetic Retinopathy Study.^21^

All specimens were harvested fresh and frozen at −20 °C until testing. Donors with lower limb amputation, prosthetic hip replacement, or a history of bone metastases were excluded from analyses. Sample hydration was maintained throughout storage and imaging.

### QCT scans

Quantitative computed tomography scans were collected of the ex vivo proximal femurs (SOMATOM Force, Siemens), beginning above the femoral head and ending 3 cm beyond the lesser trochanter (120 kVp, 600 mA, in-plane pixel size 0.68 × 0.68 mm, slice thickness 1 mm, and a standard reconstruction kernel). All femora were secured in a water bath and scanned concurrently with a calibration phantom (Mindways Software Inc.), enabling conversion from Hounsfield units into units of vBMD (mg/cm^3^).

### BCT measurements

**Figure 1:**
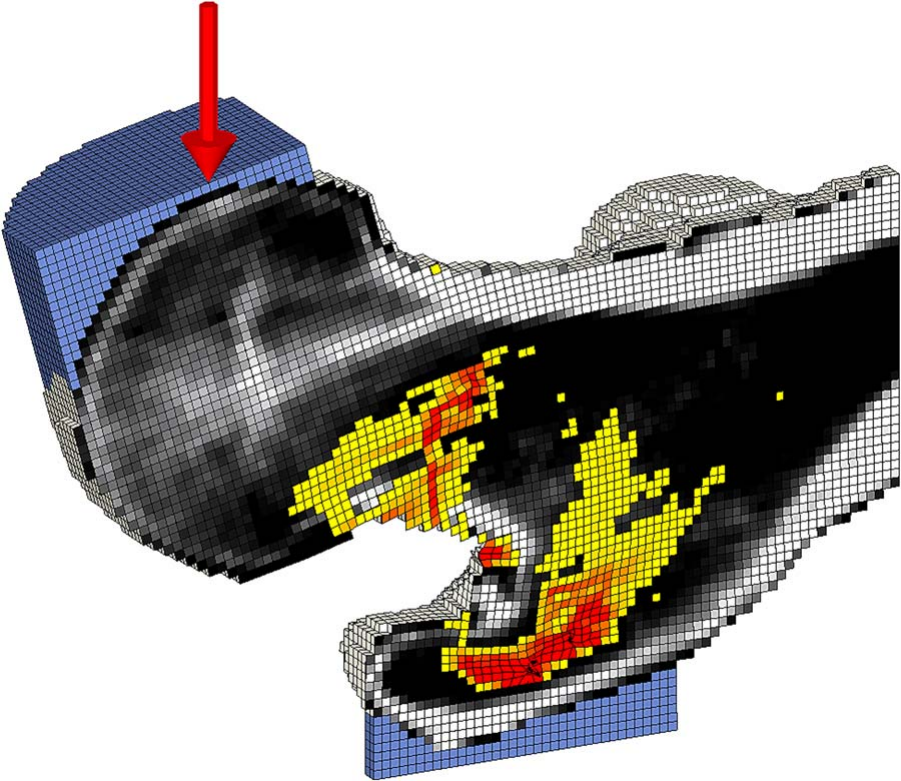
Finite element model for BCT analysis of a femur from a 68-yr-old woman with T1D, showing the results of virtual stress testing for a sideways fall. The distribution of BMD is shown in black and white. Red- and yellow-colored areas denote regions of failed bone tissue, with red indicating a higher degree of failure. BCT, biomechanical computed tomography; T1D, type 1 diabetes.

Biomechanical computed tomography analyses were performed by a single analyst at a central core laboratory (O. N. Diagnostics), which was blinded to group code. The analyses used VirtuOst (version 2.5, O. N. Diagnostics), a BCT software that is FDA-cleared for diagnostic and monitoring purposes. Biomechanical computed tomography-based bone strength at the hip and DXA-equivalent aBMD from the proximal femur were calculated from all CT scans. Details of the BCT method are described in detail elsewhere.^6^ In brief, the CT scans were calibrated to BMD, segmented, and converted into a finite element model composed of 1.0 mm-sized cubic 8-node brick elements. Each element was assigned elastic and failure properties based on empirical relations obtained from biomechanical testing of human cadaveric bone specimens.^22–25^ Then, each femur was aligned in a standard orientation simulating a sideways fall configuration^26^ and high rate of loading^7^^,^^27^ (Figure 1). BCT-based bone strength (force at 4% deformation) was calculated in 2 ways: (1) femoral strength, defined as the strength incorporating both bone geometry and bone density distribution, and (2) geometric strength, a measure of how femoral external geometry influences strength independent of the bone density. The main outcome was femoral strength, where material properties were assigned to each element based on calibrated vBMD values. For geometric strength, all intra- and inter-femoral bone density effects were removed, and an arbitrary reference density and uniform material properties were assigned to each element.^28^ By comparing geometric strength measures across all femora, the only variable is bone geometry; thus, geometric strength reflects how femoral external geometry influences strength for a sideways fall loading configuration.

To provide insight into the bone’s mechanical competence relative to its density, a strength-to-density ratio was defined by dividing each donor’s femoral *strength* by their integral volumetric bone *density* (vBMD). Additionally, a load-to-strength ratio was defined as the ratio of the *load* or fall force applied to the femoral *strength*. For each donor, the fall force was estimated as the load applied to the hip in a sideways fall from standing height using donor-specific body mass and height information.^29^ These strength metrics were calculated to reflect different biomechanical aspects of bone strength, providing a comprehensive understanding of mechanical competence in individuals with diabetes. Each strength measure offers a distinct physiological or clinical context, as outlined below.

- *Femoral strength* incorporates both the external geometry and density distribution of the bone, using voxel-specific vBMD to assign material properties. This metric provides a clinically validated and physiologically relevant estimate of femoral strength in a sideways fall configuration.^6^
- *Geometric strength* applies uniform material properties across the bone while preserving its geometry. This measure isolates the effect of hip morphology on strength.
- *Strength-to-density ratio* contextualizes the bone’s mechanical competence relative to its vBMD. As bone density alone is not a reliable indicator of fracture risk in T1D or T2D,^5^ this ratio emphasizes how femoral strength metrics (which incorporate geometry and density distribution) can reveal deficits in mechanical integrity that are not apparent from density measurements alone.
- *Load-to-strength ratio* compares the estimated load applied during a sideways fall (which incorporates the individual’s height and weight) to the bone’s failure load. A higher ratio suggests that the applied load exceeds the bone’s capacity, indicating a high fracture risk. Prior studies have shown this metric to be predictive of hip fracture in both men and women.^29^

### Medical image analysis framework measurements

**Figure 2:**
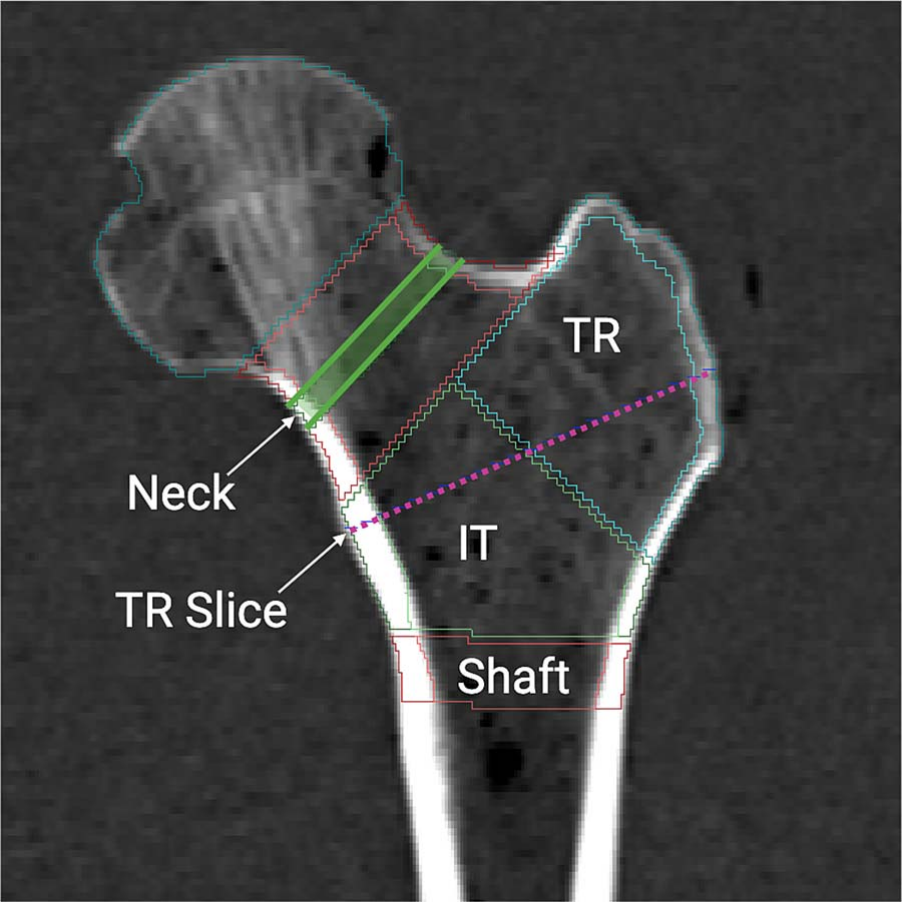
Volumes of interest (VOIs) analyzed in the proximal femur by MIAF-Femur. Note that the femoral neck VOI is shown in the shaded green box. IT, intertrochanter; TR, trochanter.

The medical image analysis framework (MIAF)–Femur software (version 7.1.0H) was used to measure volumetric bone density and morphology from the QCT scans. The details of femur segmentation and analysis by MIAF-Femur have been described previously.^30^ Briefly, a 3D segmentation technique using local adaptive thresholds and morphological detection delineated the periosteal and endosteal surfaces of the proximal femur. Based on this segmentation, volumes of interest (VOIs) of the total hip, femoral neck, trochanter, and proximal shaft were calculated based on anatomical landmarks and relative to an anatomic coordinate system centered at the smallest cross section of the femoral neck (Figure 2). The borders between VOIs were determined automatically based on anatomical landmarks and the anatomic coordinate system.^30^ The femoral neck VOI was centered at the narrowest cross section of the femoral neck and 5 mm in height (shown as the green box, Figure 2). Each VOI was separated into integral, cortical, and trabecular compartments for which vBMD (mg/cm^3^) and volume (cm^3^) were determined. Within the femoral neck VOI, the minimum overall CSA (cm^2^) was quantified. Similarly, in the trochanter slice, defined as the intertrochanteric region along the bisector of the neck–shaft angle (Figure 2), the overall trochanter CSA (cm^2^) was measured. Finally, the proximal shaft average cortical thickness (mm) was quantified.

### Statistical analyses and sample size justification

Based on analyses of bone strength of men and women with and without hip fracture,^26^ a sample size of 18 per group was chosen to provide a power of 80% at a significance level of 5% for detecting an effect size of 24%. Thus, our sample size of 19-23 specimens per group is sufficient to evaluate differences between individuals with diabetes and controls.

Each outcome was compared for group differences by an analysis of variance (ANOVA), analysis of covariance (ANCOVA), or Fisher’s exact test, as appropriate. As trends were similar between men and women (see Supplementary files), data were pooled, and all outcomes were adjusted for sex, height, and weight. Multiple comparisons were controlled using Tukey’s HSD post hoc tests, except for T-scores, fall load, and the load-to-strength ratio. T-score, fall load, and load-to-strength ratio were subject to group comparisons using a one-way ANOVA and Tukey’s HSD post hoc tests. To determine whether the lower femoral strength in individuals with diabetes was solely attributable to smaller bone size or reflected additional skeletal deficits, we conducted an additional ANCOVA where femoral strength was used as the dependent variable, constructed for geometric strength. Statistical analyses were performed using R (version 4.4.2, Vienna, Austria), with the significance level for all tests set to α ≤ .05. Graphs were made using the GraphPad Prism software (version 10.4.2, GraphPad Software). Data are presented as mean ± SD or adjusted mean (SE), as appropriate; reported percentage differences are between group means.

## Results

### Characteristics of the specimen donors

The T1D group included 13 women and 10 men with an average age at death of 79 ± 8 yr (range: 68-92 yr), the T2D group included 11 women and 10 men with an average age at death of 78 ± 7 yr (range: 63-88 yr), and the control group consisted of 11 women and 8 men with an average age at death of 79 ± 9 yr (range: 60-94) (Table 1). Age, height, and sex distribution were similar between groups (*p* > .32). BMI was significantly greater in the T2D group compared to T1D (*p* = .023), and there was a non-significant trend toward higher values in T2D compared to control (*p* = .108). The leading cause of death, cardiovascular disease, was the same in all groups (53% of control; 48% of T1D; 29% of T2D); the other most prevalent causes of death included respiratory issues, anoxia, and kidney disease.

**Table 1:** Donor characteristics for non-diabetic control, T1D, and T2D groups.

**Characteristic**	**Control** *N* = 19	**T1D** *N* = 23	**T2D** *N* = 21	**T1D vs control**	**T2D vs control**	**T1D vs T2D**
**Sex, female**	11 (58%)	13 (57%)	11 (52%)	-	-	-
**Age (years)**	79 (9)	79 (8)	78 (7)	-	-	-
**Height (m)**	1.66 (0.10)	1.68 (0.11)	1.67 (0.09)	-	-	-
**Weight (kg)**	71 (13)	72 (14)	80 (11)	-	-	-
**BMI (m/kg^2^)**	25.9 (4.4)	25.2 (3.3)	28.5 (4.3)	0.844	0.108	**0.023**
**T1D history**						
** Duration of T1D (years)**	-	67 (6)	-	-	-	-
** Age at diagnosis (years)**	-	12 (6)	-	-	-	-
** HbA1c (%)**	-	7.6 (1.08)	-	-	-	-
** Nephropathy**	-	5 (22%)	-	-	-	-
** Neuropathy**	-	21 (91%)	-	-	-	-
** Retinopathy**	-	15 (65%)	-	-	-	-
** Multiple complications^a^**	-	16 (70%)	-	-	-	-

Data presented as mean (SD); *n* (%). Analysis by one-way ANOVA followed by Tukey HSD post hoc where appropriate. Boldface indicates significant difference.

a Multiple complications defined as 2 or more of nephropathy, neuropathy, and retinopathy.

The T1D group had an average disease duration of 67 ± 6 yr and a mean age at diagnosis of 12 ± 6 yr. Individuals with T1D patients had a wide range of glucose control, with a serum HbA1c ranging from 5.0% to 9.6% (mean: 7.6%). A majority (70%) of T1D patients had multiple diabetic microvascular complications, defined as the presence of 2 or more of: nephropathy, neuropathy, or retinopathy. In the T2D group, disease duration, glycemic control, and diabetic complication status were unknown. Glycemic control and status of possible diabetic microvascular complications were also unknown in the control group.

### aBMD and bone strength by BCT

**Figure 3:**
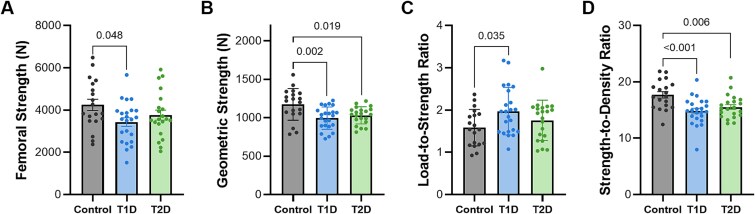
Bone strength measures by finite element analysis (FEA) demonstrated lower femoral strength (A) in T1D vs control. Geometric strength (B), reflecting bone geometry alone, was lower in both T1D and T2D vs control. The load-to-strength ratio (C) was greater in T1D but not different in T2D compared to controls, while the strength-to-density ratio (D) was lower in both T1D and T2D relative to controls. Data are presented as adjusted means (adjusted for sex, height, and weight), except for load-to-strength ratio, which is unadjusted. Error bars represent standard error, and individual data points are shown. *p*-values for Tukey HSD post-hoc tests.

Areal BMD and T-scores at the total hip and femoral neck, as well as prevalence of osteoporosis, were not statistically different between groups (*p* > .165; Table 2). When incorporating bone geometry and bone density distribution, femoral strength was significantly lower in T1D compared to control (−19%, *p* = .048; Figure 3). Although there was lower femoral strength in T2D compared to controls (−11%), the difference was not significant (*p* = .379). When femoral strength for each donor was normalized by bone density, the strength-to-density ratio was lower in T1D (−17%; *p* < .001) and T2D (−13%; *p* = .006) compared to controls, suggesting that diabetic bones were mechanically weaker than what their vBMD would predict (Figure 3). When considering only bone structure by equating tissue properties between groups, geometric strength was lower in both T1D (−15%, *p* = .002) and T2D (−13%, *p* = .019) compared to controls (Figure 3). Furthermore, although fall force was not different between groups (*p* = .345), the load-to-strength ratio was greater in the T1D cohort compared to controls (+25%, *p* = .035), suggesting greater risk of hip fracture following a fall. In contrast, the load-to-strength ratio was not different between T2D and control groups (*p* = .507; Figure 3).

**Table 2:** Biomechanical computed tomography measurements.

**Characteristic**	**Control** *N* = 19	**T1D** *N* = 23	**T2D** *N* = 21	**T1D vs control**	**T2D vs control**	**T1D vs** **T2D**
**BMD**						
** Femoral neck aBMD (g/cm^2^)**	0.66 (0.03)	0.60 (0.03)	0.65 (0.03)	-	-	-
** Femoral neck T-score^a^**	−1.26 (1.17)	−1.78 (1.36)	−1.01 (1.49)	-	-	-
** Total hip aBMD (g/cm^2^)**	0.76 (0.04)	0.72 (0.03)	0.74 (0.03)	-	-	-
** Total hip T-score^a^**	−0.65 (1.62)	−1.02 (1.78)	−0.43 (1.78)	-	-	-
**Osteoporosis^a^^,^^b^**	3 (16%)	8 (35%)	3 (14%)	-	-	-
**Bone strength**						
** Femoral strength (N)**	4310 (253)	3480 (236)	3830 (248)	**0.048**	0.379	0.581
** Geometric strength (N)**	1190 (37)	1010 (35)	1040 (36)	**0.002**	**0.019**	0.789
** Strength-to-density ratio**	17.8 (0.48)	14.7 (0.45)	15.5 (0.47)	**<.0001**	**0.006**	0.441
** Fall load (N)^a^**	5965 (889)	6045 (1065)	6366 (773)	-	-	-
** Load-to-strength ratio^a^**	1.58 (0.43)	1.97 (0.56)	1.76 (0.48)	**0.035**	0.507	0.324

Data presented as ANCOVA adjusted means (SE) (adjusted for sex, height, and weight), unless otherwise noted. Boldface indicates significant difference in Tukey HSD post hoc test.

a Unadjusted mean (SD) or *n* (%).

b Osteoporosis defined as T-score ≤−2.5 for the lower value of femoral neck and total hip BMD T-scores.

### vBMD, volume, and geometry by MIAF

While vBMD measures in all anatomical regions were not statistically different between groups, femora from the diabetic groups were smaller compared to controls (Table 3). In both T1D and T2D, femoral neck integral volume (−8%, *p* ≤ .035 for both) and minimum CSA (−8%, *p* ≤ .032 for both) were smaller compared to controls. Femoral neck trabecular volume was 9% smaller in both T1D (*p* = .078) and T2D (*p* = .063) compared to controls, although these differences did not reach statistical significance. In T1D, the smaller volume and CSA were accompanied by a narrowing in the anterior-posterior diameter (−6%; *p* = .028). In the trochanter, while the volume was similar between groups, the CSA was smaller in T1D (−7%, *p* = .012) and T2D (−8%, *p* = .007) compared to controls. In the femoral shaft, integral volume was significantly lower in T2D compared to controls (−9%, *p* = .014) and was also 7% lower in T1D (*p* = .087), although this difference was not statistically significant.

**Table 3:** Medical image analysis framework measurements.

**Characteristic**	**Control** *N* = 19	**T1D** *N* = 23	**T2D** *N* = 21	**T1D vs control**	**T2D vs control**	**T1D vs** **T2D**
**Total hip**						
** Integral BMD (mg/cm^3^)**	232 (11.3)	226 (10.5)	235 (11.1)	-	-	-
** Tb.BMD (mg/cm^3^)**	93.1 (7.81)	85.8 (7.29)	89.4 (7.66)	-	-	-
** Ct.BMD (mg/cm^3^)**	586 (17.1)	571 (15.9)	597 (16.7)	-	-	-
** Integral Vol (cm^3^)**	90 (2)	86 (2)	86 (2)	-	-	-
** Tb.Vol (cm^3^)**	57.8 (1.6)	54.8 (1.5)	54.2 (1.5)	-	-	-
** Ct.Vol (cm^3^)**	20.3 (0.5)	19.8 (0.5)	19.5 (0.5)	-	-	-
**Femoral neck**						
** Integral BMD (mg/cm^3^)**	270 (14)	258 (13)	280 (14)	-	-	-
** Tb.BMD (mg/cm^3^)**	98 (10)	91 (9)	99 (10)	-	-	-
** Ct.BMD (mg/cm^3^)**	610 (20)	576 (19)	623 (20)	-	-	-
** Integral Vol (cm^3^)**	4.14 (0.09)	3.81 (0.08)	3.82 (0.09)	**0.020**	**0.035**	0.995
** Tb.Vol (cm^3^)**	2.43 (0.08)	2.2 (0.07)	2.18 (0.07)	0.078	0.063	0.977
** Ct.Vol (cm^3^)**	1.09 (0.03)	1.02 (0.02)	1.05 (0.025)	-	-	-
** Tb.Vol/Ct.Vol**	2.2 (0.09)	2.16 (0.08)	2.08 (0.09)	-	-	-
** Minimum CSA (cm^2^)**	8.22 (0.17)	7.57 (0.16)	7.58 (0.17)	**0.021**	**0.032**	0.999
** SI diameter (mm)**	35.0 (0.44)	34.3 (0.41)	34.2 (0.43)	-	-	-
** AP diameter (mm)**	28.7 (0.48)	27.0 (0.45)	27.5 (0.47)	**0.028**	0.178	0.742
**Trochanter**						
** Integral BMD (mg/cm^3^)**	204 (11)	198 (10)	193 (10)	-	-	-
** Tb.BMD (mg/cm^3^)**	94 (8)	84 (8)	86 (8)	-	-	-
** Ct.BMD (mg/cm^3^)**	479 (17)	469 (16)	467 (17)	-	-	-
** Integral Vol (cm^3^)**	36.6 (1.1)	35 (1.1)	34.1 (1.1)	-	-	-
** Tb.Vol (cm^3^)**	23.5 (0.8)	22.2 (0.8)	22.1 (0.8)	-	-	-
** Ct.Vol (cm^3^)**	7.98 (0.29)	7.78 (0.27)	7.07 (0.28)	-	-	-
** CSA (cm^2^)**	18.9 (0.3)	17.5 (0.3)	17.3 (0.3)	**0.012**	**0.007**	0.930
**Shaft**						
** Integral BMD (mg/cm^3^)**	357 (17)	354 (15)	383 (16)	-	-	-
** Ct.BMD (mg/cm^3^)**	782 (18)	761 (17)	794 (17)	-	-	-
** Integral Vol (cm^3^)**	11.6 (0.3)	10.8 (0.3)	10.5 (0.3)	0.087	**0.014**	0.654
** Ct.Vol (cm^3^)**	4.00 (0.10)	3.75 (0.09)	3.86 (0.10)	-	-	-
** Ct.Th (mm)**	3.00 (0.09)	2.95 (0.08)	3.1 (0.09)	-	-	-

Data presented as ANCOVA adjusted means (SE) (adjusted for sex, height, and weight). Boldface indicates significant difference in Tukey HSD post hoc test.

Abbreviations: AP, anterior–posterior; CSA, cross-sectional area; Ct, cortical; Ct.Th, cortical thickness; SI, superior–inferior; Tb, trabecular; Vol, volume.

To further explore whether the observed lower femoral strength in individuals with T1D was independent of bone size, we performed an ANCOVA adjusting for femoral neck CSA, sex, and weight. The overall group effect on femoral strength did not reach statistical significance (*p* = .073), although strength appeared lower in the T1D group compared to controls (*p* = .059). These findings suggest that differences in femoral strength may not be entirely explained by CSA. A separate ANCOVA on geometric strength revealed a significant group effect (*p* = .019), with post hoc testing indicating significantly lower strength in the T1D group compared to controls (*p* = .015). These results support the interpretation that skeletal robustness may be diminished in T1D beyond differences in size or stature.

## Discussion

Numerous studies have shown that individuals with diabetes have an increased risk of hip fracture^1^^,^^2^ that cannot be fully explained by low aBMD measurements from DXA,^5^ suggesting that diabetes effects bone properties beyond aBMD. To explore this, we compared femoral vBMD, geometry, and strength in older adults with T1D or T2D to age-, sex-, and race-matched non-diabetic controls of similar age, sex, and race. Although vBMD did not differ significantly across all groups, BCT-estimated strength was lower in cadaveric femora from older adults with diabetes compared to controls, due in part to smaller proximal femur geometry.

While it is known that BCT estimates of femoral bone strength are influenced by both bone geometry and density, our findings demonstrate how diabetes-associated geometric deficits influence bone strength beyond BMD. With bone geometry as the sole variable, geometric strength reflects how femoral external geometry influences strength. Both T1D and T2D had lower geometric strength compared to non-diabetic controls, implying that smaller femoral geometry led to mechanically weaker whole bones in a sideways fall loading configuration. When femoral bone density effects were incorporated in conjunction with geometry, the strength deficits were less pronounced in individuals with T2D compared to those with T1D, consistent with epidemiological findings showing that while individuals with T2D have an elevated fracture risk relative to non-diabetics, their risk is lower than that observed in individuals with T1D.^1^^,^^2^ These findings demonstrate that skeletal fragility in T1D and T2D involves, in part, smaller femoral size and highlights the importance of structural redundancy in femoral bone strength. Structural redundancy refers to the ability of a structure to retain its function without catastrophic failure, even when 1 or many parts of the structure fail, due to the presence of redundant load paths.^31^ In the femur, this concept is supported by greater CSA, favorable cortical distribution, and robust architectural design, which enable mechanical loads to be redistributed across undamaged regions. In the present study, individuals with T1D and T2D exhibited smaller femoral size, and those with T1D also demonstrated lower strength, suggesting a lower capacity redistribute mechanical loads. While we did not directly assess structural redundancy, the lower strength in T1D may reflect a diminished ability to redistribute loads, leaving the bone more vulnerable to mechanical failure. These observations underscore how compromised architectural design may weaken mechanical resilience and contribute to elevated fracture risk in diabetes, independent of bone density.

Additionally, individuals with diabetes had a lower strength-to-density ratio compared to controls, suggesting that femora from individuals with T1D and T2D were mechanically weaker than would be expected based on BMD alone. This aligns with prior reports that fracture risk is elevated in diabetes independently of BMD.^5^ As standard clinical tools like DXA rely solely on areal BMD, they may not fully capture the skeletal deficits associated with diabetes. Finite element models have previously been shown to estimate femoral strength with greater fidelity to mechanical failure than aBMD alone,^6–8^ but future studies are needed to evaluate the comparative predictive performance of these tools in a larger diabetic population.

The present ex vivo findings highlight the potential relevance of bone quality factors beyond BMD, such as bone structure, in contributing to fracture risk. In particular, the strength-to-density ratio has the potential to serve as a biomarker for evaluating bone fragility in diabetes, but its clinical utility in predicting fractures and guiding treatment requires further investigation. Taken together, these findings highlight the multifactorial nature of diabetic bone disease and the need for more comprehensive approaches to fracture risk assessment that extend beyond traditional density-based metrics.

Although estimated femoral strength was reduced in individuals with T1D, the magnitude of this difference was modest compared to the well-established hip fracture risk. Individuals with T1D face approximately a 5-fold increased risk of hip fracture compared to non-diabetics, yet the difference in femoral strength in this study was comparatively small (−20%). This discrepancy suggests that neither DXA-derived aBMD nor strength estimates fully explain the elevated fracture risk in these populations. Additional contributors, such as changes in bone material properties,^17^^,^^32–34^ increased fall risk,^4^ diabetes duration, and systemic complications,^35^^,^^36^ likely play a significant role. These findings emphasize the importance of multidimensional approaches to fracture risk assessment that integrate biomechanical, clinical, and metabolic factors in individuals with diabetes.

Our findings of geometric deficits in T1D build upon those reported in a limited number of previous studies using DXA and QCT. Specifically, we observed smaller femoral neck CSA in older adults with T1D using QCT, confirming and expanding upon earlier reports that identified deficits in femoral neck CSA in older adults with T1D from DXA hip structural analysis (HSA)–estimated geometry.^37^ It is important to note that while DXA-based HSA can be used to derive measurements of femoral geometry, HSA estimates of cross-sectional properties are based on simplified beam models that assume uniform mineral density and circular cross section at the neck.^38^ Thus, DXA-based geometric measurements do not reflect the true variability in bone structure.^38^ In contrast, QCT enables direct measurement of cross-sectional properties, accounting for variation in geometry and material distribution. In contrast to our observations of geometric impairments in T1D, a recent study using QCT reported no significant difference in femoral neck CSA between older adults (>50 yr) with longstanding T1D and non-diabetic controls.^11^ However, this prior study did observe deficits in femoral neck bone density and structure in those with microvascular disease (nephropathy or neuropathy) as well as those diagnosed with T1D before the age of 15.^11^ Other studies have similarly found that younger age at diagnosis and presence of microvascular disease affect bone outcomes in T1D.^39–41^ While we did not directly compare these sub-groups, 91% of the T1D group in the current study had microvascular disease (nephropathy or neuropathy) and 74% were diagnosed before 15 yr of age. Thus, while the effect of age at diagnosis and presence of microvascular disease were not directly assessed, the structural deficits observed in our T1D cohort may indirectly reflect these important clinical factors.

Our study also provides insight into altered hip structure in individuals with T2D despite normal to high measures of BMD compared to control. Little is known regarding altered hip structure in individuals with T2D, with some but not all previous studies reporting deficient hip structure in T2D using DXA HSA.^35^ Observations at peripheral skeletal sites have demonstrated smaller tibial and radial CSA in older men and women with T2D.^35^^,^^42^ Expanding on these previous observations, using QCT, we observed lower femoral neck volume and smaller CSA at the femoral neck and trochanter in older adults with T2D compared to controls.

Deficits in bone size and increased fracture risk in older adults with T1D and T2D may result from diabetes-induced deficiencies during growth (T1D) and impairments in the age-related geometric adaptations necessary to maintain bone strength (T1D and T2D). During growth, insulin deficiency, chronic hyperglycemia, and disruptions in the growth hormone-insulin-like growth factor axis combine to limit periosteal expansion and reduce bone size.^43^ Insulin, an anabolic hormone, is critical for promoting osteoblast activity and bone formation,^44^ and its absence in T1D directly impairs bone growth and matrix deposition. Additionally, hyperglycemia promotes the formation of advanced glycation end products (AGEs), which accumulate in bone collagen and reduce both bone quality and the mechanical signaling necessary for growth.^45^ Delayed puberty and chronic inflammation further limit peak bone mass, exacerbating the risk of fracture later in life. In healthy aging, periosteal apposition compensates for declining BMD by increasing bone CSA.^46^^,^^47^ thereby maintaining bone strength. However, smaller CSAs observed in individuals with T1D and T2D suggest that diabetes may impair this compensatory response. While bone size plays a role in overall strength, our adjusted analyses imply that skeletal fragility in T1D likely reflects broader structural deficits. The persistence of strength differences after accounting for CSA, weight, and sex points toward potential alterations in bone architecture or quality, such as disrupted distribution of cortical and trabecular bone, that may underlie compromised mechanical performance in this population.

In addition to structural changes, molecular alterations in bone tissue may also play a critical role. For example, downregulation of canonical Wnt signaling in T2D has been associated with greater AGE accumulation and lower bone strength, suggesting impaired bone formation and remodeling at the cellular level.^48^ Alterations in osteocyte mechanoregulation and signal transduction, observed in both T1D and T2D,^36^^,^^49^^,^^50^ may limit bone’s adaptation to mechanical forces, leading to sub-optimal periosteal apposition and smaller bone area. Although the exact mechanisms linking diabetes to deficits in bone size require further study, these structural impairments likely contribute to the high fracture risk observed in individuals with diabetes. Understanding factors such as the duration of T2D will provide valuable insight into the relative contributions of development and age-related deficits in bone geometry.

While bone mass and geometry are key determinants of bone strength, bone tissue material properties also play an important role. Previous work from our lab^17^^,^^51^ and others^32–34^^,^^52^^,^^53^ has demonstrated altered collagen structure, including accumulation of AGEs, as well as impaired mechanical behavior in bone tissue collected from patients with T1D and T2D. Importantly, cortical bone samples extracted from the mid-diaphysis of the current T1D cohort showed impaired ability to absorb energy during mechanical loading (ie, lower toughness), which was associated with ﻿cortical tissue mineral density, the AGE carboxy-methyl lysine, and Raman spectroscopic measures of enzymatic collagen crosslinks and glycosaminoglycan content.^17^ Thus, modest deficits in bone strength due to smaller bone geometry could be exacerbated by moderate deficits in bone material properties. Note that our BCT models of bone strength in this study assume equivalent bone density-derived material properties and thus may underestimate actual strength deficits from normal bone. While beyond the scope of the current work, studies comparing laboratory ex vivo biomechanical testing and BCT measures of bone strength could help to provide insight into how much differences in material properties influence overall bone strength in diabetes.

Our study should be interpreted in the context of its limitations. First, while clinical studies have demonstrated relationships between poor glycemic control and long disease duration to prevalent fractures in diabetes,^35^^,^^36^ we were unable to investigate the influence of these clinical factors on bone density, geometry, or strength measures due to the small sample size of our T1D cohort and lack of detailed clinical information in T2D. Further work in a larger population with greater clinical detail is needed. Second, use of tissue from the Medalist study may limit the generalizability of our results in the broader T1D population. In particular, the Medalists have extreme disease duration (>50 yr), relatively well-controlled A1c, and lower severity of traditional diabetic complications, which may not be representative of the larger T1D population. Another limitation of the current study is the lack of co-morbidity or fracture history for the commercial tissue bank donors, which could provide deeper insight into the underlying mechanisms of fragility in T1D and T2D. Additionally, we did not correct for multiple comparisons when analyzing the data. As a result, some of the findings may represent false positives due to the increased likelihood of detecting significant results by chance when making numerous comparisons, particularly given that the number of comparisons exceeded the sample size in each group. While the primary analysis pooled men and women due to generally similar trends, our sex-stratified supplementary analyses suggest that some of the observed geometric differences, particularly at the femoral neck, may be more consistent in men than in women. This raises the possibility that the skeletal impact of diabetes may differ by sex and highlights the need for future studies that are adequately powered to assess sex-specific effects. However, given the literature’s incomplete understanding of bone density, geometry, and strength in T1D and T2D, our study provides novel information by demonstrating lower bone strength beyond what would be predicted by BMD and identifies potential geometric alterations that likely contribute to skeletal fragility in older adults with diabetes. Thus, despite some limitations, our data using human cadaveric femora from donors with and without diabetes provide new insight into potential mechanisms contributing to skeletal fragility in patients with T1D and T2D.

In conclusion, we found that diabetes was associated with lower FEA-estimated geometric (T1D and T2D) and femoral (T1D) strength. These deficits were associated with smaller femoral neck and trochanter geometry. Overall, these findings may help to explain the higher fracture risk in the diabetic population. Our results suggest the importance of further investigations of femoral geometry and FEA bone strength in larger studies of skeletal fragility in T1D and T2D populations and underscore the need to consider factors beyond BMD when assessing fracture risk in diabetic populations.

## Supplementary Material

Supplementary_information_ziaf127

## Data Availability

The data underlying this article will be shared on reasonable request to the corresponding author.
