# Supplementary material for: Femora from adults with type 1 or type 2 diabetes have lower bone strength and smaller hip geometry
Source: JBMR Plus. 2025 Jul 30;10(3):ziaf127. doi: 10.1093/jbmrpl/ziaf127 (PMC12908684; doi:10.1093/jbmrpl/ziaf127)
Supplement: Supplementary_information_ziaf127 [file supplementary_information_ziaf127.docx]

## **Supplementary Information**

### Methods

Each outcome was stratified by sex and compared for group differences by an analysis of covariance (ANCOVA) or Fisher’s exact test, as appropriate. All outcomes were adjusted for height, and weight. Multiple comparisons were controlled using Tukey’s HSD post-hoc tests, except for T-scores, fall load, and the load-to-strength ratio. Since the fall load was calculated using the subject’s height and weight, metrics using fall load were subject to group comparisons using a one-way ANOVA and Tukey’s HSD post-hoc tests. Given the small sample size (n=8-13 per group), results should be interpreted cautiously due to limited power and potential overfitting.

### Results

Table S 1: BCT measurements stratified by sex

|  | **Female** | | | | | | |  | **Male** | | | | | | |
| --- | --- | --- | --- | --- | --- | --- | --- | --- | --- | --- | --- | --- | --- | --- | --- |
|  | **Control** | **T1D** | **T2D** | **ANCOVA** | **T1D vs. Control** | **T2D vs. Control** | **T1D vs. T2D** |  | **Control** | **T1D** | **T2D** | **ANCOVA** | **T1D vs. Control** | **T2D vs. Control** | **T1D vs. T2D** |
| **Characteristic** | N = 11 | N = 13 | N = 11 | **p-value** |  |  |  |  | N = 8 | N = 10 | N = 10 | **p-value** |  |  |  |
| **BMD** |  |  |  |  |  |  |  |  |  |  |  |  |  |  |  |
| Femoral Neck aBMD (g/cm^2^) | 0.60 (0.031) | 0.55 (0.030) | 0.58 (0.033) | 0.584 | - | - | - |  | 0.71 (0.052) | 0.63 (0.045) | 0.75 (0.046) | 0.190 | - | - | - |
| Femoral Neck T-score *^1^* | -1.68 (1.00) | -2.32 (0.84) | -1.77 (0.95) | 0.197 | - | - | - |  | -0.68 (1.19) | -1.07 (1.60) | -0.17 (1.56) | 0.411 | - | - | - |
| Total Hip aBMD (g/cm^2^) | 0.65 (0.037) | 0.64 (0.035) | 0.65 (0.039) | 0.979 | - | - | - |  | 0.87 (0.057) | 0.78 (0.051) | 0.88 (0.052) | 0.353 | - | - | - |
| Total Hip T-score *^1^* | 0.66 (0.13) | 0.61 (0.13) | 0.67 (0.14) | 0.568 | - | - | - |  | 0.56 (1.34) | 0.19 (1.75) | 0.71 (1.62) | 0.758 | - | - | - |
| Osteoporosis*^1,2^* | 2 (18%) | 6 (46%) | 3 (27%) | 0.351 | - | - | - |  | 1 (13%) | 2 (20%) | 0 (0%) | 0.481 | - | - | - |
| **Bone Strength** |  |  |  |  |  |  |  |  |  |  |  |  |  |  |  |
| Femoral Strength (N) | 3300 (220) | 2800 (210) | 2900 (230) | 0.201 | - | - | - |  | 5400 (480) | 4100 (430) | 5000 (430) | 0.130 | - | - | - |
| Geometric Strength (N) | 1000 (49) | 850 (47) | 920 (52) | **0.050** | **0.041** | 0.360 | 0.582 |  | 1400 (52) | 1200 (46) | 1200 (47) | **0.002** | **0.004** | **0.004** | 0.995 |
| Fragile Bone Strength *^1,3^* | 5 (45%) | 9 (69%) | 8 (73%) | 0.457 | - | - | - |  | 1 (13%) | 3 (30%) | 1 (10%) | 0.579 | - | - | - |
| Strength-to-Density Ratio | 15 (0.53) | 13 (0.50) | 13 (0.55) | **0.002** | **0.004** | 0.013 | 0.971 |  | 21 (0.83) | 17 (0.73) | 19 (0.75) | **0.004** | **0.003** | 0.155 | 0.168 |
| Fall Load (N) *^1^* | 5,373 (617) | 5,274 (453) | 5,796 (496) | 0.052 | - | - | - |  | 6,780 (425) | 7,047 (726) | 6,992 (470) | 0.592 | - | - | - |
| Load-to-Strength Ratio*^1^* | 1.68 (0.39) | 2.06 (0.52) | 1.97 (0.42) | 0.121 | - | - | - |  | 1.45 (0.47) | 1.86 (0.62) | 1.52 (0.43) | 0.195 | - | - | - |
| Data presented as ANCOVA adjusted means (SE) (adjusted for height and weight), unless otherwise noted. | | | | | | |  |  |  |  |  |  |  |  |  |
| Boldface indicates significant difference in ANCOVA or Tukey HSD post-hoc test. | | | |  |  |  |  |  |  |  |  |  |  |  |  |
| *^1^* Unadjusted mean (SD) or n (%). | | | | | | |  |  |  |  |  |  |  |  |  |
| *^2^* Osteoporosis defined as T-score ≤ -2.5 for the lower value of femoral neck and total hip BMD T-scores. | | | | | | |  |  |  |  |  |  |  |  |  |
| *^3^* Fragile bone strength defined as unadjusted femoral strength ≤ 3,000 N for women, ≤ 3,500 N for men. | | | | | | |  |  |  |  |  |  |  |  |  |

Table S 2: MIAF measurements stratified by sex

|  | **Female** | | | | | | |  | **Male** | | | | | | |
| --- | --- | --- | --- | --- | --- | --- | --- | --- | --- | --- | --- | --- | --- | --- | --- |
|  | **Control** | **T1D** | **T2D** | **ANOVA** | **T1D vs. Control** | **T2D vs. Control** | **T1D vs.  T2D** |  | **Control** | **T1D** | **T2D** | **ANOVA** | **T1D vs. Control** | **T2D vs. Control** | **T1D vs.  T2D** |
| **Characteristic** | N = 11 | N = 13 | N = 11 | **p-value** |  |  |  |  | N = 8 | N = 10 | N = 10 | **p-value** |  |  |  |
| **Total Hip** |  |  |  |  |  |  |  |  |  |  |  |  |  |  |  |
| Integral BMD (mg/cm^3^) | 210 (12) | 220 (12) | 220 (13) | 0.809 | - | - | - |  | 250 (18) | 230 (16) | 260 (17) | 0.346 | - | - | - |
| Tb.BMD (mg/cm^3^) | 76 (9.2) | 71 (8.7) | 70 (9.6) | 0.890 | - | - | - |  | 110 (13) | 98 (12) | 110 (12) | 0.618 | - | - | - |
| Ct.BMD (mg/cm^3^) | 540 (19) | 560 (18) | 580 (19) | 0.366 | - | - | - |  | 630 (26) | 570 (23) | 630 (23) | 0.148 | - | - | - |
| Integral Vol (cm^3^) | 71 (2.1) | 67 (2) | 72 (2.2) | 0.204 | - | - | - |  | 110 (3.6) | 110 (3.2) | 100 (3.3) | 0.083 | - | - | - |
| Tb.Vol (cm^3^) | 44 (1.5) | 41 (1.4) | 45 (1.6) | 0.112 | - | - | - |  | 74 (2.9) | 69 (2.5) | 65 (2.6) | 0.088 | - | - | - |
| Ct.Vol (cm^3^) | 16 (0.59) | 16 (0.56) | 17 (0.62) | 0.767 | - | - | - |  | 25 (0.83) | 24 (0.73) | 23 (0.74) | 0.241 | - | - | - |
| **Femoral Neck** |  |  |  |  |  |  |  |  |  |  |  |  |  |  |  |
| Integral BMD (mg/cm^3^) | 260 (16) | 260 (15) | 270 (16) | 0.958 | - | - | - |  | 270 (22) | 250 (19) | 300 (19) | 0.130 | - | - | - |
| Tb.BMD (mg/cm^3^) | 80 (12) | 82 (12) | 91 (13) | 0.818 | - | - | - |  | 120 (16) | 95 (15) | 110 (15) | 0.624 | - | - | - |
| Ct.BMD (mg/cm^3^) | 590 (22) | 580 (21) | 600 (24) | 0.887 | - | - | - |  | 620 (31) | 560 (27) | 670 (28) | **0.041** | 0.326 | 0.538 | **0.033** |
| Integral Vol (cm^3^) | 3.5 (0.12) | 3.1 (0.11) | 3.3 (0.12) | 0.140 | - | - | - |  | 4.9 (0.12) | 4.5 (0.11) | 4.3 (0.11) | **0.005** | **0.032** | **0.004** | 0.552 |
| Tb.Vol (cm^3^) | 1.9 (0.091) | 1.7 (0.087) | 1.9 (0.096) | 0.371 | - | - | - |  | 3.1 (0.12) | 2.7 (0.1) | 2.5 (0.1) | **0.005** | 0.053 | **0.004** | 0.379 |
| Ct.Vol (cm^3^) | 1 (0.026) | 0.9 (0.024) | 0.94 (0.027) | **0.012** | **0.009** | 0.166 | 0.504 |  | 1.2 (0.043) | 1.2 (0.038) | 1.2 (0.038) | 0.587 | - | - | - |
| Tb.Vol/Ct.Vol | 1.9 (0.099) | 1.9 (0.094) | 2 (0.1) | 0.737 | - | - | - |  | 2.6 (0.14) | 2.4 (0.12) | 2.1 (0.12) | **0.031** | 0.479 | **0.026** | 0.193 |
| Minimum CSA (cm^2^) | 6.9 (0.23) | 6.3 (0.22) | 6.6 (0.24) | 0.135 | - | - | - |  | 9.8 (0.25) | 8.9 (0.22) | 8.6 (0.22) | **0.005** | **0.033** | **0.004** | 0.551 |
| SI Diameter (mm) | 32 (0.63) | 31 (0.6) | 31 (0.66) | 0.655 | - | - | - |  | 39 (0.62) | 38 (0.55) | 38 (0.56) | 0.439 | - | - | - |
| AP Diameter (mm) | 27 (0.7) | 25 (0.67) | 26 (0.74) | 0.199 | - | - | - |  | 32 (0.6) | 29 (0.53) | 29 (0.54) | **0.013** | **0.037** | **0.015** | 0.862 |
| **Trochanter** |  |  |  |  |  |  |  |  |  |  |  |  |  |  |  |
| Integral BMD (mg/cm^3^) | 180 (13) | 180 (12) | 170 (14) | 0.912 | - | - | - |  | 230 (16) | 210 (14) | 220 (14) | 0.707 | - | - | - |
| Tb.BMD (mg/cm^3^) | 69 (9.9) | 59 (9.4) | 62 (10) | 0.764 | - | - | - |  | 120 (14) | 110 (12) | 120 (12) | 0.868 | - | - | - |
| Ct.BMD (mg/cm^3^) | 430 (23) | 460 (22) | 450 (24) | 0.727 | - | - | - |  | 520 (23) | 470 (20) | 500 (21) | 0.291 | - | - | - |
| Integral Vol (cm^3^) | 30 (1.2) | 28 (1.2) | 29 (1.3) | 0.524 | - | - | - |  | 45 (2) | 42 (1.7) | 40 (1.8) | 0.167 | - | - | - |
| Tb.Vol (cm^3^) | 19 (0.8) | 17 (0.76) | 19 (0.84) | 0.314 | - | - | - |  | 29 (1.5) | 27 (1.3) | 26 (1.4) | 0.289 | - | - | - |
| Ct.Vol (cm^3^) | 6.5 (0.4) | 6.4 (0.38) | 6.2 (0.42) | 0.855 | - | - | - |  | 9.8 (0.39) | 9.1 (0.34) | 8.1 (0.35) | **0.015** | 0.387 | **0.012** | 0.134 |
| CSA (cm^2^) | 17 (0.36) | 15 (0.34) | 16 (0.38) | **0.007** | **0.005** | 0.19 | 0.334 |  | 22 (0.52) | 20 (0.46) | 19 (0.47) | **0.009** | 0.065 | **0.007** | 0.482 |
| **Shaft** |  |  |  |  |  |  |  |  |  |  |  |  |  |  |  |
| Integral BMD (mg/cm^3^) | 320 (18) | 340 (17) | 360 (18) | 0.264 | - | - | - |  | 390 (27) | 360 (23) | 420 (24) | 0.201 | - | - | - |
| Ct.BMD (mg/cm^3^) | 730 (21) | 730 (20) | 780 (22) | 0.234 | - | - | - |  | 830 (24) | 780 (21) | 830 (21) | 0.195 | - | - | - |
| Integral Vol (cm^3^) | 11 (0.32) | 9.5 (0.3) | 9.2 (0.34) | **0.011** | **0.040** | **0.016** | 0.848 |  | 13 (0.48) | 12 (0.42) | 12 (0.43) | 0.250 | - | - | - |
| Ct.Vol (cm^3^) | 3.4 (0.11) | 3.2 (0.1) | 3.3 (0.11) | 0.518 | - | - | - |  | 4.6 (0.16) | 4.2 (0.14) | 4.5 (0.14) | 0.123 | - | - | - |
| Ct.Th (mm) | 2.7 (0.083) | 2.8 (0.079) | 2.8 (0.087) | 0.437 | - | - | - |  | 3.3 (0.16) | 3.1 (0.14) | 3.5 (0.14) | 0.199 | - | - | - |
| Data presented as ANCOVA adjusted means (SE) (adjusted for height and weight). Boldface indicates significant difference in ANCOVA or Tukey HSD post-hoc test. | | | |  |  |  |  |  |  |  |  |  |  |  |  |
| Notes: Tb = trabecular; Ct = cortical; BMD = bone mineral density; Vol = volume; Ct.Th = cortical thickness; CSA = cross-sectional area; SI = superior-inferior; AP = anterior-posterior | | | | | | | | | | | | |  |  |  |
